# The Current Evidence Regarding COVID-19 and Pregnancy: Where Are We Now and Where Should We Head to Next?

**DOI:** 10.3390/v13102000

**Published:** 2021-10-05

**Authors:** Theodoros Kalampokas, Anna Rapani, Maria Papageorgiou, Sokratis Grigoriadis, Evangelos Maziotis, George Anifandis, Olga Triantafyllidou, Despoina Tzanakaki, Spyridoula Neofytou, Panagiotis Bakas, Mara Simopoulou, Nikolaos Vlahos

**Affiliations:** 1 Assisted Conception Unit, Second Department of Obstetrics and Gynecology, Aretaieion Hospital, Medical School, National and Kapodistrian University of Athens, 76, Vasilisis Sofias Avenue, 11528 Athens, Greece; kalamp@yahoo.com (T.K.); mariapap96@windowslive.com (M.P.); sokratis-grigoriadis@hotmail.com (S.G.); vagmaziotis@gmail.com (E.M.); triantafyllidouolga@gmail.com (O.T.); dtzanakaki@gmail.com (D.T.); spinik@yahoo.gr (S.N.); pbakas74@gmail.com (P.B.); nfvlahos@gmail.com (N.V.); 2 Laboratory of Physiology, Medical School, National and Kapodistrian University of Athens, 75, Mikras Asias, 11527 Athens, Greece; rapanianna@gmail.com; 3 Department of Histology and Embryology, Faculty of Medicine, University of Thessaly, 41500 Larisa, Greece; ganif@uth.gr

**Keywords:** pregnancy, COVID-19, complications, delivery, neonatal health, transmission, breastfeeding, vaccination

## Abstract

Despite the volume of publications dedicated to unraveling the biological characteristics and clinical manifestations of SARS-CoV-2, available data on pregnant patients are limited. In the current review of literature, we present an overview on the developmental course, complications, and adverse effects of COVID-19 on pregnancy. A comprehensive review of the literature was performed in PubMed/Medline, Embase, and Cochrane Central databases up to June 2021. This article collectively presents what has been so far reported on the identified critical aspects, namely complications during pregnancy, delivery challenges, neonatal health care, potential routes of viral transmission, including vertical transmission or breastfeeding, along with the risks involved in the vaccination strategy during pregnancy. Despite the fact that we are still largely navigating uncharted territory, the observed publication explosion in the field is unprecedented. The overwhelming need for data is undoubtable, and this serves as the driver for the plethora of publications witnessed. Nonetheless, the quality of data sourced is variable. In the midst of the frenzy for reporting on SARS-CoV-2 data, monitoring this informational overload is where we should head to next, considering that poor quality research may in fact hamper our attempts to prevail against this unparalleled pandemic outbreak.

## 1. Introduction

A global effort to investigate the pathophysiological mechanisms of SARS-CoV-2 has been noted since the beginning of the current pandemic. The noted explosion of interest in investigating COVID-19 to provide data, map the virus’ biological identity, and guide clinicians towards prevention and management strategies [1] is unparalleled. Fertility and reproduction have been in the spotlight of recent publications, since SARS-CoV-2 targets female reproductive organs that express its main receptor ACE2 [2,3]. Hitherto, studies have described the symptomatology, the developmental course and the complications characterizing the COVID-19 disease, while identifying certain patient characteristics that constitute risk factors for manifesting poor outcomes. Nonetheless, the effect of COVID-19 on pregnancy, leading to a unique state of different human physiology, has yet to be fully elucidated [4]. 

The limited and contradicting data on pregnant patients have resulted in a lack of established guidelines. Coupled by the fact that the vast discrepancies in management may be a strong indication of poor standards and fast track publication policies, these circumstances cause uncertainty both for the patients and clinicians. The current review of literature provides an all-inclusive overview of the published studies concerning the impact of COVID-19 on several aspects of pregnancy. The complications and adverse effects on maternal health status are presented, along with data on the optimal delivery method for pregnant patients who have tested positive for COVID-19. The subsequent neonatal health and potential risk of vertical transmission or a potential viral transmission during breastfeeding are further discussed. The debated matter of vaccination policy during pregnancy is presented, as well as the latest available data in this field of interest. The aim of this review is to collectively present evidence on the impact of COVID-19 on several aspects of pregnancy and discuss how data contribute to the scientific progress during this pandemic. The wealth of information is overwhelming, yet fails to provide definitive conclusions. What becomes apparent is the challenge in navigating this maze of publications, while the variable quality of data sourced adds another level of complexity. 

## 2. Materials and Methods

A comprehensive review of the literature was performed in PubMed/Medline, Embase, and Cochrane Central databases up to June 2021. Literature screening was performed employing a combination of Medical Subject Headings (MeSH) terms and keywords, including: “2019 novel coronavirus pandemic”’; “COVID-19”; “severe acute respiratory syndrome coronavirus 2”; “SARS-CoV-2”; “coronavirinae’’; “coronavirus infection’’; “pregnancy”; “pregnancy outcome”; “pregnancy complications”; “neonatal outcomes”; “perinatal outcomes’’; “delivery”; ‘’labor’’; “vertical transmission”; “mother to fetus transmission”; “breastfeeding”; “vaccination”; “vaccines”; “vaccination safety’’. The search was limited to full-length manuscripts published in English in international peer-reviewed journals. Original research articles describing studies performed in humans as well as review papers were sourced. In order to provide an all-inclusive analysis of the current evidence, no specific inclusion and exclusion criteria regarding study selection process were employed. Regarding type of study, different types of studies were considered eligible to be included in this review, namely prospective and retrospective observational and interventional studies, randomized controlled trials, case reports and case series, as well as systematic reviews and meta-analysis. From the articles retrieved in the first round of search, additional references were identified by manual citation mining. Following literature assessment, authors categorized the sourced studies according to the specific topic of research investigated in five categories, namely: 1. studies investigating complications of COVID-19 reported during pregnancy; 2. studies investigating labor-related challenges in pregnant women infected by SARS-CoV-2; 3. studies investigating neonatal and perinatal outcomes in neonates born from COVID-19 positive mothers; 4. studies aiming to address the possible mechanisms of SARS-CoV-2 vertical transmission; 5. studies examining breastfeeding-related concerns; and 6. studies debating vaccination efficacy and safety during pregnancy and lactation. A critical analysis of these aspects was performed in order to provide an all-inclusive overview of the current evidence.

## 3. Complications of COVID-19 Reported in Pregnancy

Prior to discussing the complications of COVID-19 during pregnancy, a primary factor that seems to exert a substantial impact on the manifestation of the disease is the timing of viral exposure. The association between the risk of viral transmission and certain stages of pregnancy remains vague. The cases of two pregnant women who were found to be positive for SARS-CoV-2 during the first weeks of pregnancy have been reported [5]. In the second trimester of their pregnancy, both underwent amniocentesis for the evaluation of the existence of SARS-CoV-2 RNA, as well as for an assessment of antibodies in amniotic fluid samples. Despite the negative results, the concern of a potential in utero transmission during the first trimester merits further investigation. Concerning cases of COVID-19 infection during the second trimester of pregnancy, interesting conclusions are proposed. Tang et al. revealed two second trimester pregnancies that tested positive for COVID-19 [6]. At the time of delivery, both women had a negative SARS-CoV-2 RNA test in throat swab samples, but elevated titles of antibodies. Both babies were healthy and throat swabs tested negative. IgG antibodies levels were elevated in both cases, due to transmission from the mother, despite lacking any sign indicative of acute infection. One case of a COVID-19 positive pregnant woman who delivered in the second trimester has been also reported [7], with no evidence supporting the potential transmission of the virus. 

Regarding the complications and their severity attributed to the diagnosis of COVID-19, a special interest has been noted in unveiling the factors contributing to adverse health outcomes and to the deterioration of health status. The range of clinical manifestations described in cases of pregnant patients diagnosed with COVID-19 includes mild flu-like symptoms to the onset of severe pneumonia. Fever and cough constitute the most frequent symptoms described in pregnant women, while myalgia, shortness of breath, sore throat, nasal congestion, diarrhea, headache, and chills are further contributing to the symptomatology (Table 1). The clinical course of the disease in pregnant and non-pregnant women has been investigated by Wang et al. Interestingly, a milder clinical course was described in pregnant women, along with a higher rate of asymptomatic cases and a reduced duration of hospitalization [8]. Based on the observations of 43 pregnant women who tested positive for SARS-CoV-2, 29 of them presented to the hospital suffering from COVID-19 symptoms, while the remaining 14 were asymptomatic. In two cases, severe complications involving respiratory distress syndrome were developed [9].

Another study including symptomatic pregnant women showcased the need for closer and meticulous monitoring of these patients when they are older than thirty-five years, and while characterized by at least one comorbidity, namely obesity, gestational diabetes, or hypertension [34]. Eight out of the seventy pregnant women with a severe or critical diagnosis of COVID-19 that were included in the study by Blitz et al. required intubation, with subsequent documentation of two deaths [55]. To emphasize the significance of obesity, a 10% ICU hospitalization rate has been reported in pregnant women with increased body mass index, attributed as the sole statistically significant factor contributing to this outcome [35]. Moreover, in a UK cohort study involving four hundred twenty-seven pregnant women with COVID-19, one in ten women were hospitalized and required respiratory support in ICU, while 70% of the patients were reported with increased BMI, 40% were >35 years old, and a third had comorbidities [15]. A case report of a 41-year-old obese and diabetic pregnant patient who manifested respiratory failure and required mechanical ventilation [53], as well as the case of two asymptomatic patients who developed symptoms of an upper respiratory tract infection following labor have been documented. In the last report, it should be emphasized that both of these two patients were obese and diabetic, while one of them had a history of chronic hypertension and asthma [50].

Besides the investigation of obesity as a factor contributing to the manifestation of severe complications, the role of the week of gestation during the time of diagnosis has been assessed and concluded as a potential parameter affecting severity of the disease. According to a case-control study evaluating ICU admissions and the requirement of respiratory support among pregnant and non-pregnant women diagnosed with COVID-19, pregnant women diagnosed when they were over the 20th week mark of gestation were at a higher risk of severe adverse outcomes, in comparison to the non-pregnant group. Amongst the two groups of patients, other comorbidities or obesity were not found to be remarkably differentiated [56]. A study including 64 women with severe or critical manifestations of COVID-19 demonstrated that all of the patients who experienced critical symptoms were >24th week of gestation at the time of the initial symptoms. Contrary to the abovementioned study, in this report comorbidities were documented, including pulmonary conditions and cardiac diseases in 25% and 17%, respectively [32].

Little is known in regard to the cardiovascular complications of coronavirus disease 2019 in pregnancy. Differentiating between postpartum cardiomyopathy and COVID-19-related cardiomyopathy in infected pregnant women is challenging. The case of a young pregnant woman who exhibited signs of heart failure with pulmonary edema following cesarean section has been presented in the literature [57]. Coagulopathy is considered to be associated with COVID-19, which in turn may result in the onset of further complications, including deep vein thrombosis [58]. The case of an obese, young, pregnant woman who developed ovarian vein thrombosis while being diagnosed with COVID-19 has been documented in the literature [59]. Another report on an obese, young, pregnant woman showcased an event of pulmonary embolism despite her being administered with prophylactic anticoagulation protocol [44]. The case of a pregnant woman who tested positive for COVID-19 has been published, showcasing the onset of venous sinus thrombosis following symptoms of headache and hemiparesis. As concluded in that case report, in the presence of suspected hypercoagulability and atypical features, venous sinus thrombosis should be considered in the differential diagnosis for patients with COVID-19 [60]. Acute pancreatitis constitutes a rare complication of primary COVID-19 infection, as presented in a case report describing a patient who was diagnosed while being hospitalized due to COVID-19-related pneumonia. The patient exhibited signs of improvement postpartum and was further discharged home [10]. Moreover, the impact of SARS-CoV-2 on the neurological system has been emphasized, as the virus’s neurotropism potential has been in the spotlight of research. Some cases of Guillain–Barre Syndrome associated with COVID-19 have been recently revealed [13,61].

Considering the impact of SARS-CoV-2 infection during pregnancy on maternal and neonatal morbidity and mortality, recent published data from large epidemiological studies warrant great interest and thus should be highlighted. One of the largest cohorts that have been published so far is the INTERCOVID study [62]. This is a multicenter multinational cohort study, including 2130 women from 43 institutions and 18 countries, from March to October 2020. The authors of this study investigated to what extent SARS-CoV-2 infection during pregnancy could increase the risk of adverse maternal and neonatal outcomes in comparison to pregnant women without COVID-19. In total, 706 pregnant women positive for SARS-CoV-2 were included in the study. To minimize the respective bias, for each of the study participants, the authors included two matched not-infected women, serving as controls. In total, 1424 not-infected women were allocated to the control group. The study and the control groups were matched according to the stage of pregnancy, the type and stage of delivery, as well as the level of patient care received. The primary outcome measures were the incidence of adverse pregnancy, neonatal, and perinatal outcomes, including morbidity and mortality. Statistical analysis was performed, employing models to adjust the findings according to country, month entering study, maternal age, and medical history. Provided data indicated that SARS-CoV-2 infection during pregnancy is strongly associated with adverse pregnancy outcomes, including pre-eclampsia/eclampsia, severe infections, intensive care unit admission, and medically induced preterm labor. The risk of adverse neonatal and perinatal outcomes, such as severe neonatal morbidity and severe perinatal morbidity and mortality, also presented to be statistically significantly increased. In addition, COVID-19-related symptoms, such as fever and shortness of breath were associated with increased risk of severe maternal and neonatal complications. Interestingly, even the asymptomatic women presented with an increased risk of pregnancy complications, including pre-eclampsia and higher maternal morbidity. Infant positivity for SARS-CoV-2 was calculated to be 13%. Delivery via cesarean section but not breast feeding was associated with an increased risk of neonatal transmission [62]. Similar results are also provided from other smaller cohort studies performed in different populations worldwide, including cohorts in Spain, Turkey, India, and Iran [63,64,65,66]. These recently published data demonstrate that in comparison to the general pregnant population, COVID-19 infected pregnant women present with increased risk of adverse maternal, neonatal, and perinatal outcomes, highlighting the need for careful monitoring of pregnancies implicating COVID-19. 

On the antipode of this influx of evidence demonstrating that pregnant women may experience a more severe clinical manifestation of COVID-19, one study reported that pregnant women with comorbidities were not characterized by a higher risk of hospitalization. Moreover, in this study, non-pregnant patients more frequently reported fever, contrary to pregnant patients who frequently reported symptoms of myalgia, fatigue, and headaches [22]. On the same note, pregnant women exhibit a lower risk of developing a severe symptomatology if diagnosed with COVID-19 in comparison to the general population [16]. However, the cases describing and raising awareness on the phenomenon of maternal deaths call for cautious conclusions regarding the actual risk that pregnant women may experience. Amongst published studies referring to maternal deaths, Hantoushzadeh et al. presented the cases of nine severely affected pregnant women. Seven out of nine died due to cardiopulmonary complications, one remained intubated in the ICU, and one recovered, while it should be noted that the majority of the patients had no comorbidities [54]. 

Clinical manifestations of pregnant women that tested positive for COVID-19 should be further examined and considered when managing this cohort of patients. In an effort to evaluate the common laboratory test findings in pregnant women with COVID-19, interesting observations have been published. In the majority of these patients, a normal count of white blood cells has been reported, while lymphopenia constitutes the most common finding. In cases of women with severe symptomatology who require admission to the ICU, the lymphocyte count was found to be lower [35]. Thrombocytopenia has been described in three mild cases of pregnant patients [67]. Such a finding should be highlighted and acknowledged prior to initiating any invasive pregnancy-related procedure, such as the placement of an epidural catheter. Increased levels of alanine aminotransferase (ALT) and aspartate aminotransferase (AST), as well as elevated C-reactive protein (CRP) is present in many cases of pregnant patients. Inflammation marker levels are remarkably higher in pregnant patients who have tested positive for the SARS-CoV-2 in comparison to the non-pregnant group [8]. Regarding the computed tomography (CT) findings, pregnant women were subjected to chest CT, representing the modality of choice for early detection. The typical findings of viral pneumonia were detected similarly to the cases of non-pregnant patients [68]. These findings include decreased diffuse and ground glass opacities, patchy lung consolidation, blurred borders, and lesions merged into strips in some cases [49]. As evident in literature, severe pneumonia, tracheal intubation and artificial ventilation, as well as an emergency cesarean section were performed under general anesthesia in a 39-year-old woman diagnosed with COVID-19 at 25 weeks of gestation. This suggests the need to explore the risk of increased coronavirus disease severity during pregnancy, the impact on perinatal prognosis, as well as the management that pregnancy requires under such circumstances [69].

## 4. Challenges during Delivery of Pregnant Patients with COVID-19

Since SARS-CoV-2 transmission and COVID-19 pathophysiology remain vague, the timing and mode of delivery constitute a challenging issue. Based on current evidence, the guidelines suggest that the delivery mode should be individualized and personalized, based on the obstetric indications and the maternal–fetal status [70]. The indications for performing a C-section include prematurity, breech presentation, fetal intrauterine distress, premature rapture of membrane, arrest of descent, arrest of dilation, failed induction, decrease in the fetal heart rate, severe pre-eclampsia, history of another C-section, abnormal amniotic fluid, umbilical cord or placenta (placenta previa), and no fetal movement or no variability of fetal heart monitoring. As evident in several studies, due to the lack of data on determining the risk of intrapartum mother-to-child transmission, vaginal delivery was avoided. 

A higher risk of adverse outcomes related to delivery have been attributed to cases of pregnant women with COVID-19. More specifically, iatrogenic preterm births and C-sections are more often expected in comparison to pregnant women who tested negative for COVID-19 [71]. To add to this observation, Knight et al. reported that among the preterm births that were observed, 80% were required due to the deterioration of the maternal health status [15]. Furthermore, the rate of preterm births and C-sections among critically ill pregnant patients was notably elevated. Interestingly, as it has been voiced, 75% of critically ill pregnant women gave birth prematurely, while the 94% delivered by C-section due to the deterioration of their health status [32]. Amongst patients hospitalized in the ICU, 80% delivered via C-section [35]. Several studies report the performance of C-sections on the grounds of severely compromised maternal status, such as respiratory insufficiency and pulmonary embolism that required urgent attention and intervention [44,53,54]. Contrary to the above, there is a case of a COVID-19 positive pregnant woman in the 33rd week of gestation, for whom delivery was required in order to improve the maternal respiratory status. Following labor induction, vaginal delivery was performed while the patient was under ventilation with an impressive outcome. Therefore, the need for strict patient selection when contemplating delivery method should be prioritized, since despite the worsening respiratory status of some pregnant patients indicating the need for performing a C-section, they may still undergo an induced vaginal labor [29]. 

Whether delivery itself could ameliorate the severe effects of COVID-19 and restore maternal health status is a valid question. A study demonstrated that an improvement of the respiratory status may be observed following delivery. Nonetheless, whether the delivery mode is implicated to affect maternal status post-partum remains to be validated [72]. When concrete data concerning the risks involved in delivery method is published, clinicians should be able to establish a common strategy that will ascertain optimal obstetric and perinatal results, safeguarding both the women’s and the newborn’s safety. 

## 5. Neonates’ Health Status

No significant differences have been observed regarding the clinical course and the laboratory findings in neonates born to mothers diagnosed as positive, compared to those who have tested negative for COVID-19. The only finding of significance concerns the significantly decreased birthweight in neonates born by mothers positive for COVID-19 [7]. Moreover, Hantoushzadeh et al. have reported three cases of fetal deaths in cases of critically ill mothers [54]. The case of a newborn that tested positive for SARS-CoV-2 immediately following birth via C-section has been published. The baby manifested a severe course of the disease with tachypnea, cyanosis, and dyspnea subsequently requiring respiratory support. Both the baby and mother, who were intubated for twenty-four hours, were safely discharged home [25]. Sisman et al. described the case of a premature neonate born by a COVID-19 positive mother who developed fever, hypoxia, and neonatal respiratory distress syndrome in the second day of life, and tested positive in the throat swab test for COVID-19. It was assumed that this case constitutes a congenital infection based on the placenta findings [27]. Two severely ill premature neonates born by COVID-19 positive mothers were intubated in the neonatal intensive care unit and underwent a prolonged hospitalization [73]. In this study, an interesting point is raised with regard to the potential association between premature neonates and a more severe course of COVID-19. However, in such cases, prematurity stands as a confounder, allowing for no further extrapolations to be drawn concerning the severity of the disease in these babies. 

Regarding the reported complications observed in neonates, neonatal pneumonia, mild grunting following birth due to mild Newborn Respiratory Distress Syndrome (NRDS), tachypnea, and moaning are reported. All these cases were successfully treated, employing continuous positive airway pressure ventilation [40,46,74]. Zhu et al. described one neonate, delivered at a gestational age of 34+5 weeks, who developed shortness of breath, moaning, and thrombocytopenia along with abnormal liver function. Due to multiple organ failure and disseminated intravascular coagulation, its death was reported on the ninth day of admission. However, another case presenting with a common symptomatology was successfully treated by employing respiratory support, and recovered fifteen days later [49]. In a study by Vivanti et al., a neonate, whose mother tested positive for SARS-CoV-2, developed neurological symptoms. Three days following birth, it exhibited irritability, poor feeding, axial hypertonia, and opisthotonos, whereas a sample of cerebrospinal fluid was collected and further tested negative for the virus. The neonate gradually recovered and was finally discharged eighteen days later [51]. An overview of neonates’ health status born by mothers that were positive for COVID-19 is depicted in Table 2. 

## 6. Delineating the Phenomenon of Vertical Transmission

A crucial concern that challenges obstetricians is whether a transplacental transmission could occur in cases of pregnant patients diagnosed with COVID-19. The placenta constitutes a specialized organ, vital for the development of the fetus as well as for the protection of the fetus. However, as depicted in the literature, many bacteria or viruses, such as cytomegalovirus, human immunodeficiency virus, and rubella virus, could cross the placenta barrier and infect the fetus [76]. Despite the fact that many placenta pathologies have been described [77], transplacental transmission and its frequency still remain a controversial topic of scientific interest. 

In a study performed by Yu et al., the nucleic acid test for the throat swab of one neonate was positive for SARS-CoV-2 thirty-six hours following birth [37]. However, in the abovementioned case, intrauterine tissue samples, including placenta and cord blood, were detected as negative, rendering the hypothesis of a potential intrauterine vertical transmission vague. Another report describes a case of vertical transmission from the asymptomatic mother to the baby. The molecular detection of SARS-CoV-2 in mother’s blood at delivery and in the neonatal nasopharyngeal confirmed the infection [78]. Alwardi et al. reported a case of preterm triplets born by a coronavirus positive pregnant woman. All of them tested positive from the nasopharyngeal swab drawn twenty hours following birth, while one of the triplets required nasal ventilation for eight hours [30]. In a study by Khan et al., seventeen swab samples were tested for SARS-CoV-2, out of which two were positive. However, the viral nucleic acid test of placenta, cord blood, or amniotic fluid were not tested to confirm whether intrauterine vertical transmission has occurred [40]. Along the same lines, Alzamora et al. confirmed infection on a neonate’s nasopharyngeal swab sixteen hours following birth. Nonetheless, amniotic fluid, cord blood, or placental tissue samples were not tested in order to investigate the presence of the virus [53]. Interestingly, Marzollo et al. revealed the case of a possible congenital COVID-19 infection. A full-term neonate who was delivered vaginally by a positive tested mother demonstrated respiratory and gastrointestinal symptoms soon after birth [31]. 

A proven case of transplacental transmission of SARS-CoV-2 from a pregnant woman affected by COVID-19 during the third trimester of pregnancy has been published [51]. The nasopharyngeal and rectal swabs, as well as placenta samples were collected and further tested positive for SARS-CoV-2 by employing RT-PCR. It should be noted that the viral load was significantly higher in the placental tissue than in amniotic fluid or in maternal or neonatal blood. Moreover, the first study to report persistent placental infection of SARS-CoV-2 and its congenital transmission has been recently published. As mentioned, the transmission is associated with hydrops fetalis and intrauterine fetal demise during the stages of early pregnancy. In this study, the case of a pregnant asymptomatic woman in the first trimester who tested positive for COVID-19 at the 8th week of gestation is presented. At 13 weeks of gestation, the patient tested negative, however viral RNA was detected in the placenta, suggesting that the SARS-CoV-2 had crossed the placental barrier, and viral RNA was then detected in the amniotic fluid [79].

The risk of infection during vaginal delivery further perplexes any attempts to delineate the vertical viral transmission process. The increased risk of mother to infant transmission by intrapartum exposure to amniotic fluid, sac, or membranes has been demonstrated by a study examining eleven placental and membranal swabs for the detection of the virus [80]. Fenizia’s et al. findings also support the in utero transmission of SARS-CoV-2. The virus’s genome was isolated in cord plasma, which is exclusively fetal [75]. The second case that was described supports an in utero transmission, due to the state of an infected placenta and the presence of antibodies in cord blood. While the first case refers to a patient with a severe course of COVID-19 disease, the second one refers to a patient with mild symptoms. Therefore, establishing a connection between the risk of transmission and the severity of the disease cannot be concluded. 

Delineating whether a vertical viral transmission may occur is a crucial and urgent matter. Not only due to the fact that it could compromise the fetal health status, but further—as demonstrated by the following studies—a vertical transmission could be indicative of severe adverse effects during pregnancy that will require a clinician’s special attention and management strategy. The case of a patient at 22nd week of gestation, whose pregnancy was complicated by severe pre-eclampsia resulting in termination has also been described. The placenta findings demonstrated the presence of SARS-CoV-2, localized predominantly at the maternal–fetal interface of the placenta. This viral invasion of the placenta should be emphasized, as it may constitute a crucial factor of severe morbidity in pregnant patients [20]. Moreover, another report presented the case of a pregnancy with normal development, which following the mother’s COVID-19 infection exhibited severe complications including critical blood flow in the fetal umbilical artery, fetal growth restriction (first percentile), hydropericardium, right ventricular hypertrophy, and intraventricular hemorrhage. As a result, the baby was prematurely delivered in the 26th week, resulting in its death due to asystole. Test results indicated that a vertical transmission of SARS-CoV-2 had occurred from mother to the fetus [11]. A preterm infant born to a mother with severe COVID-19 pneumonia has been reported in the literature. The amniotic fluid tested positive for SARS-CoV-2, while the newborn exhibited signs of an early-onset infection with SARS-CoV-2, suggesting the possibility of vertical transmission [12]. On the other hand, a patient with monochorionic–diamniotic twins being diagnosed with COVID-19 at 15 weeks of gestation has been described. Following severe complications, namely stage II twin–twin transfusion syndrome, subchorionic hematoma, *Escherichia coli* bacteremia, and septic shock, a preterm delivery was initiated at 21 weeks of gestation. Amniotic fluid and placenta were negative for SARS-CoV-2, arguing the case against transplacental transmission following a second-trimester infection [81]. Another issue of great importance that remains unknown is whether the intervillositis that was described in the abovementioned study was provoked by COVID-19 infection, since this finding is known to be associated with miscarriage, fetal growth restriction, or pre-eclampsia. Similarly, in another study, miscarriage of preterm twins born by a mother who experienced COVID-19 symptoms two weeks prior to delivery has been reported. SARS-CoV-2 was detected in placenta samples and amniotic fluid, nonetheless it was absent in the amniotic sac. Moreover, the placenta histology showed signs of chronic intervillositis. All these findings are consistent with the hypothesis of vertical transmission and further reinforce the potential link between miscarriages and COVID-19 infection [26]. Despite the fact that placental COVID-19 infection has been reported in some cases during the second and third trimester, no documentation of such phenomenon has been published considering the first trimester of pregnancy. However, it has been recently indicated that in the placenta and fetal organs examined from an early pregnancy miscarriage in a COVID-19 positive mother, SARS-CoV-2 nucleocapsid protein, viral RNA, and particles consistent with coronavirus have been detected. These findings validated for the first time that congenital SARS-CoV-2 infection could be feasible during the first trimester of pregnancy. This constitutes an alarming observation that should be considered when clinicians assess and manage pregnant patients, since the risk of adverse perinatal outcomes in cases of infection during the early pregnancy stage could be detrimental [82]. A report investigating the impact of SARS-CoV-2 on a twin pregnancy diagnosed with infection at the third trimester of gestation, identified a pattern of cytokines including IL1-Ra, IL-9 G-CSF, IL-12, and IL-8 that were differently expressed in both twins, suggesting that the SARS-CoV-2-induced cytokine storm is not impaired during the placental passage [83]. On the other hand, in an analysis of nineteen placentas of COVID-19 positive women, a variety of pathologies were described, albeit the absence of chronic intervillositis was validated [84]. Smithgall et al. compared fifty-one third trimester placentas of women positive for COVID-19, with twenty-five placentas of pregnant women testing negative. Although the first group exhibited signs of maternal–fetal vascular malperfusion, no definite association of SARS-CoV-2 could be concluded [18]. Therefore, it has become evident that the absence of a typical placental pathology indicates the need for further studies, in order to investigate the possibility of placenta infection.

Since IgG and IgM antibody testing for SARS-CoV-2 became widely available, new criteria were established in order to determine a potential intrauterine viral transmission. Maternal IgG is passively transferred across the placenta from mother to fetus, while this transmission primarily occurs during the last trimester of gestation. On the other hand, IgM cannot be transferred through the placenta due to its larger size [85]. Therefore, elevated levels of IgM antibodies could probably indicate in utero infection, assuming that the virus was transmitted through the placenta and IgM antibodies were then produced by the infant. Dong et al. studied an infant delivered by a mother with COVID-19 via C-section [45]. Although the viral nucleic acid tests of the neonate’s nasopharyngeal swab and the breastmilk sample were both negative, IgM and IgG antibody levels were elevated in the infant’s blood sample collected two hours post birth. In another study, two neonates had elevated IgM antibodies and five neonates exhibited elevated IgG antibodies [1]. Gao et al. proposed the case of a potential intrauterine transmission of coronavirus, based on the elevated IgM antibodies in neonate’s serum, attributed to the mother’s exposure to the virus six weeks prior to delivery [86]. The case of a pregnant patient with COVID-19, whose pregnancy was complicated with RhD alloimmunization, makes for an interesting observation [87]. Due to fetal anemia, three intrauterine transfusions were performed by the 30th week of gestation. Following the procedure, IgM and IgG antibodies measured in the fetal blood sample were negative, indicating no signs of virus transmission from the mother to the fetus. In the 32nd week of gestation, due to maternal complications including progressive shortness of breath, a cesarean section was performed. Amniotic fluid, cord blood, and the neonate’s throat swab tested negative for SARS-CoV-2, while the mother’s nasopharyngeal swab was positive for COVID-19. Consequently, data regarding antibodies against severe acute respiratory syndrome coronavirus 2 are therefore limited. More serologic data should be accumulated in controlled, meticulously designed studies with control groups, in order to investigate the neonatal exposure to the virus.

**Figure 1:**
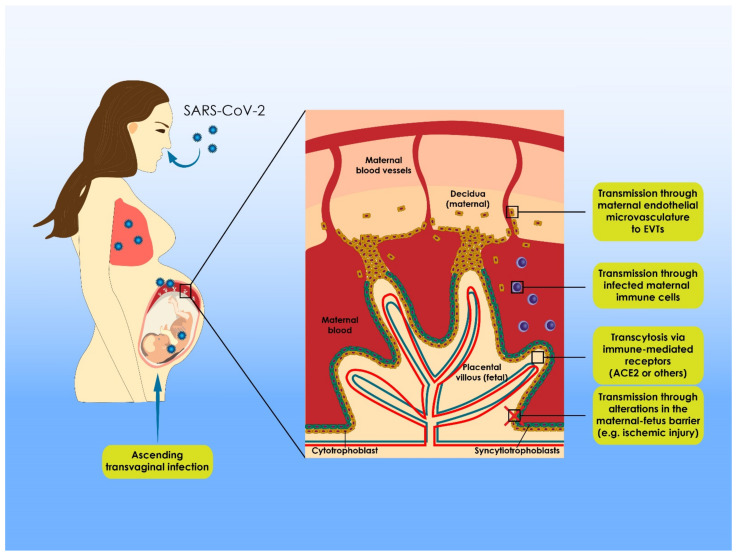
Suggested pathways for SARS-CoV-2 transmission from infected mothers to fetuses during pregnancy. Limited data are available regarding the role of placenta in SARS-CoV-2 infection, and thus the mechanisms of possible vertical transmission are still poorly understood. Considering the current knowledge, five possible infection routes have been proposed. Following infection of the mother, SARS-CoV-2 virions spread throughout the body via maternal circulation, finally reaching the maternal–fetal interface. According to the first suggested mechanism, transmission of SARS-CoV-2 could be achieved through maternal endothelial microvasculature to extravillous trophoblasts (EVTs) and other placenta cells expressing angiotensin converting enzyme 2 (ACE2) receptors. According to the second mechanism, SARS-CoV-2 virions could stimulate immune response in the maternal–fetal interface, inducing accumulation of maternal immune cells in the infected area, such as macrophages. Maternal immune cells could then be infected, as these cells express ACE2 receptors. Following this, the infected maternal immune cells could infiltrate the placenta and transmit the virus to the fetal cells (cell-to-cell transmission). The third proposed mechanism involves possible alterations in the maternal–fetal barrier, including ischemic injury and increased release of inflammatory regulators. These alterations lead to increased SARS-CoV-2 virions’ permeability throughout the placenta, leading finally to virions spreading in the fetal environment. According to the fourth proposed mechanism, both syncytiotrophoblasts and their rupture could directly be infected by virion transcytosis, mediated via immune receptors, including ACE2 and Fc (FcR). Finally, fetal infection originating from ascending vaginal infection has also been proposed.

The dynamic changes of antibodies against coronavirus in neonates born by COVID-19 positive mothers have been described [88]. Fifteen out of twenty-four neonates had increased levels of IgG antibodies and six had increased IgM levels, while none developed respiratory symptoms and all tested negative for the presence of the virus. The levels of the IgG antibodies which may reflect the passive immunity [76] in neonates decreased slower in neonates who exhibited elevated IgM antibodies. As the literature suggests, maternal IgG antibodies remain in neonate’s serum for approximately six months, providing them with essential protection from infections [89]. The findings of Dong et al. are extremely interesting, since they emphasize a rapid decrease in the levels of IgG antibodies against SARS-CoV-2 in neonates’ blood in a time frame of less than one and a half months. This indicates the potential increased risk of COVID-19 infection for the neonates [23]. Table 3 and Figure 1 portray the current evidence on the potential routes of vertical transmission, as presented in the included studies herein.

## 7. Risks Entailed in Breastfeeding

Apart from intrauterine vertical transmission and infection during delivery, the issue of breastfeeding along with the viral transmission risks entailed raise concerns for obstetricians. In order to evaluate this hypothesis, breastmilk samples have been assessed for RNA presence, while in some cases IgM and IgG antibodies against SARS-CoV-2 were further measured. The cases of two mothers positive to SARS-CoV-2 who were lactating have been described [90]. Breastmilk samples of one mother, who experienced mild symptoms of COVID-19, were positive for four continuous days. The newborn exhibited symptoms relevant to the respiratory system and tested positive for SARS-CoV-2, while the transmission route could not be assessed. 

In the study by Lang et al., several breastmilk samples were repeatedly tested following delivery in order to measure viral RNA. In total, all results were negative and mothers were encouraged to breastfeed following a fourteen day isolation period [42]. In another study, the case of direct breastfeeding by a mother who tested positive for SARS-CoV-2 has been described. Breastmilk samples were continuously tested for viral presence, while antibodies against SARS-CoV-2 were measured. SARS-CoV-2 nucleic acid was not detected in the breast milk, whereas antibodies were detected in both the mother’s serum and milk. Therefore, this case provided a confirmation that the viral transmission via breastmilk alone might be extremely rare, rendering breastfeeding a safe feeding method for an infant [91]. On the same note, the study by Salvatore et al. reports a cohort of neonates born by mothers positive to SARS-CoV-2, and follows the results of rooming in and breastfeeding up to one month following birth [21]. All the neonates tested negative for SARS-CoV-2, either immediately following birth or fourteen days later. This indicates that rooming in and breastfeeding may be safe when the necessary precautions are taken into consideration, including hand hygiene and use of surgical masks.

It is widely known that breastfeeding provides infants with protection against infections, mainly via secretory IgA antibodies [92]. Dong et al. report the presence of IgG and IgA antibodies in breast milk, which seem to trigger the immune protection in the neonate [23]. Another study reports the case of a premature neonate born by a healthy asymptomatic mother, who developed symptoms and tested positive for coronavirus three days following birth. Although the newborn was breastfed, and milk was later tested positive for COVID-19, but the newborn did not develop any symptomatology [93]. The potential protective role of maternal antibodies against COVID-19 should be taken into consideration, in order to assess the risk-benefit of breastfeeding [75]. More recently, a study including 55 newborns of SARS-CoV-2-positive mothers reported that no viral infection was detected in the neonates who received unpasteurized breast milk following birth. All infants were breastfed at home and remained SARS-CoV-2 negative. These findings may provide an insight regarding the safety of breastfeeding [94].

## 8. Vaccination Debate

It is well established in clinical practice that the majority of vaccines are permitted during pregnancy, as their benefit often outweighs the potential risk entailed [95]. Therefore, a few observations have been reported concerning women included in vaccine clinical trials who experienced an unanticipated pregnancy. Pharmaceutical companies developing COVID-19 vaccines exclude pregnant individuals from their clinical trials. Moreover, due to the limited available information on the safety and efficacy of vaccines during pregnancy, it has been proposed to avoid conceiving for weeks to months following vaccination [96]. Furthermore, since mRNA vaccines do not utilize an adjuvant nor do they constitute live vaccines, the American College of Obstetricians and Gynecologists (ACOG) along with the Society for Maternal-Fetal Medicine (SMFM) have stated that “these vaccines should not be withheld from pregnant and breastfeeding women”. Nonetheless, the FDA has yet to issue any guidelines delineating employment of COVID-19 vaccines during pregnancy, while the emergency authorization use (EAU) letters that mRNA vaccines have received label pregnant women as “a population of interest” [97].

Interestingly, the main point of concern is that vaccination may initiate a cascade of symptoms, namely headache, fatigue, chills, and most importantly fever. Maternal fever during the third trimester of pregnancy has been linked to an increased risk of developing neonatal birth defects [98]. The transplacental transfer of SARS-CoV-2 antibodies following maternal vaccination in the third trimester may pose as a strong indicator that when a mother receives a COVID-19 vaccine, the neonate is protected to an extent. The role of the timing of vaccination when considering the level of protection that the transferred autoantibodies may offer has yet to be decoded. As proposed, additional longitudinal follow-up studies of a larger scale that will strictly monitor vaccinated patients are required to correlate pregnancy and neonatal outcomes with maternal vaccination. In the meantime, patients’ own preference along with their healthcare provider’s suggestion should determine whether vaccination should be considered [99]. As a prerequisite, the evaluation of individualized risk factors should be undertaken [100]. 

## 9. Discussion

Management of pregnant patients during these unprecedented times encompasses numerous aspects of investigation. It is prudent to thoroughly consider the risks and concerns entailed in all phases and stages of pregnancy to identify the COVID-19-related parameters that may jeopardize the end goal of a healthy “take-home baby”. Crucial aspects are still under investigation, including the concerns raised on the route of transmission from the mother to the fetus, along with the developmental course and severity of complications of COVID-19 during pregnancy [101]. Could the physiological changes that occur during pregnancy involving the cardiovascular, respiratory, and coagulation system, coupled by a COVID-19 diagnosis establish an increased morbidity risk? Thus far, data suggest that pregnant patients with severe SARS-CoV-2 infection are at an increased risk of perinatal complications compared to asymptomatic pregnant patients [14]. Most studies reporting on the consequences of COVID-19 infection concern the third trimester of pregnancy. Moreover, the role of certain risk factors, such as obesity, should be evaluated. Management and monitoring of pregnant patients of different profiles may differ, hence we are called to thoroughly profile pregnant patients to ascertain appropriate management. 

Our intention was to provide an all-inclusive overview on what is thus far known and reported on COVID-19 and pregnancy, highlighting current concerns and areas of special interest. Undoubtably, this has been an extraordinary year for medicine that has shaped and transformed scientific research. An accelerating pace in COVID-19-related research has been noted, as there are thousands of publications dedicated to COVID-19 and pregnancy. Nonetheless, this wealth of published data presents with amplified weaknesses in research methodology, and current publication policies that could prognosticate the challenges researchers may face in the future [102]. The high heterogeneity observed among the studies led the authors to refrain from performing a systematic review on this topic. It is well documented that systematic reviews provide an objective analysis of the relevant evidence, in contrast to the narrative reviews which are characterized by subjectivity. Nonetheless, the quality of evidence provided by a systematic review is strongly associated with the quality of the included studies. Considering the lack of robust data on COVID-19 aspects, a systematic review could potentially, at present, fail to serve its own purpose, which is reaching a robust conclusion and may further present the risk of confusing the readership. However, the authors acknowledge that the quality of a narrative review may be improved by following a systematic approach for literature evaluation, and have herein adopted an effective search strategy, using specific key-words and data assessment strategy [103].

The critical analysis of the current data—constituting the aim of this comprehensive review—results in certain aspects becoming clear. It becomes evident that a risk assessment is vital for pregnant women who are diagnosed as positive for COVID-19. Especially when comorbidities are present, the complications may be severe, demanding close monitoring similar to a high-risk pregnancy. Up until now, studies’ findings in the literature are collectively pointing to the direction that certain identified risk factors in pregnant patients indicate a higher risk of complications. Studying the inevitable heterogeneity of pregnant patients diagnosed with COVID-19 who are included in current and future studies will further unravel additional risk factors that play a crucial role in the developmental course of the disease. Regarding the delivery mode, a vast number of studies were characterized by missing outcome data and selection report bias, therefore, assessing the short-term and long-term repercussions of opting for vaginal delivery or c-section is still under investigation. For the time being, the lack of sufficient data and high-quality methodology leads clinicians to assess the risks and benefits based on the individual’s health status alone. Interestingly, regardless of delivery mode, most of the cases of newborns in the literature seem to respond well and achieve health status restoration. However, 2019-nCoV infection may exert severe adverse effects on newborns, such as neonatal pneumonia, prematurity, neonatal respiratory distress syndrome, and even neonatal death. The deafening heterogeneity amongst studies along with the preliminary nature of the published data dictate that interpretation of results sourced hitherto should be performed with caution. Despite the increasing number of publications dedicated to this topic, unbiased conclusions cannot be drawn due to the inadequacy of good quality evidence.

Moving on to the matter of viral transmission, delineating the routes of transmission is of paramount significance, that will enable us to optimize the management of COVID-19 pregnant patients and their delivery options. Clinical research should soon be qualified to provide definitive evidence on the mechanisms and the physiology entailed when considering the possibility of COVID-19 vertical transmission. Thus far, no safe conclusions can be drawn with respect to the routes of transmission, due to the lack of consistency in the evidence reporting on mother to newborn transmission. In regard to breastfeeding, larger cohort studies are essential to confirm the rarity of perinatal transmission when strict safety measures are applied. What is more, the physiology mechanisms entailed in the protection that breastfeeding appears to offer should be further evaluated. Current publications investigating the matter of breastfeeding in COVID-19 patients raise the concern stemming from the heterogeneity of patients examined. Following the recommendations from World Health Organization, there is no indication to stop breastfeeding as advocated by the vast majority of concordant published data, as evident in literature thus far. The concerns for the safety of breastfeeding should be validated by larger studies, since isolating the newborn and the mother as a precautionary protection measure while lacking conclusive evidence may affect the newborn’s emotional attachment. In an effort to draw conclusions on the heated topic of vaccination, data thus far suggest that considering the risks associated with COVID-19 in pregnant patients, vaccination should be encouraged. Contemplating the wide application of vaccination in this group of patients, follow-up and assessment in both the mother and the fetus from cases that have already received the vaccine will allow us to draw a safer conclusion in the future [104]. 

The responsibility of the scientific community should be highlighted. Research topics unrelated to COVID-19 may come second best while as estimated 80% of clinical trials have been interrupted during the past few months in an effort to dedicate available resources to this crusade against SARS-CoV-2 [102]. Thus, where do we go from here? The pandemic is new and old at the same time. Due to the rapid emergence of new data, several studies are now outdated—despite the recent dates of publication—and novel ones appear to present the minor updated findings. Perhaps the eagerness to share, report, and publish on “everything COVID-19 related” may not in fact be as beneficial. The question raised is, what is the true impact of this overwhelming volume of publications? Does it assist physicians to achieve their goal in optimizing their clinical practice during this pandemic? Or could it be that this vast influx of data stands as a backpedal, hindering scientific progress? This is a time to ponder on whether “any data is good data” and the answer may be no. Instead of being fast-tracked and prioritized, it should be recommended for value and standards to be rigorously assessed in COVID-19 data, in the context of “what it offers”. It is of paramount significance to ensure that publications of research on COVID-19 are more regulated. Until this informational overload that bombards the scientific community is effectively managed, clinicians should abide by an ethical, moral, and legal duty towards their patients regarding shared decision making, while prioritizing patients’ safety [105].

## 10. Conclusions

There is an urgency to define the optimal strategy to manage pregnant patients diagnosed with SARS-CoV-2. It becomes evident that the huge volume of articles published since the beginning of the pandemic is impressive. However, this is coupled by a lack of conclusive theses, due to discrepancies and heterogeneity which is almost alarming, albeit anticipated. Extrapolated hypotheses emerge daily, perhaps lacking filtering mechanisms to exclude data that may be of poor quality. What becomes clear is that this overload of data fails to lead to robust conclusions, but rather paradoxically reflects the current unknown situation we are still facing nearing two years into the pandemic. The research field as a whole may be affected by the inevitable urgency to address COVID-19, albeit failing to lead to robust data and clear conclusions. Based on the current contradicting findings referring to the role of COVID-19 on the various phases of pregnancy, and the lack of robust original studies, no safe conclusions can be drawn. To prevail against this unparalleled pandemic outbreak, high quality information is needed. Perhaps it is time for the scientific community to suggest a strategy to monitor and control COVID-19-related data flow in every discipline, to ascertain a successful and timely response to this ongoing crisis.

## Figures and Tables

**Table 1:** An overview of the reported symptomatology in pregnant patients diagnosed with COVID-19, as described in the included studies.

Study	No of Pregnant Women	Trimester/ Gestation	No of Asymptomatic Women	Fever	Cough	Dyspnea	Myalgia	Headache	Diarrhea	Other
[10]	1	33 w	-	0	1	1	1	0	0	Nausea, vomiting, acute pancreatitis
[11]	1	21 w	-	1	1	0	0	0	0	Anosmia, ageusia
[12]	1	32 w	-	1	1	0	1	0	0	Anorexia, nausea
[13]	1	20 w	-	0	1	0	0	0	0	Acroparaesthesia, bilateral lower extremity weakness, dysphonia, dysphagia, Guillain–Barré syndrome
[14]	1219	37.7 w (median)	579	214	414	230	232	188	63	Nasal stiffness, chills, anosmia, fatigue, sore throat, nausea
[15]	427	29–38 w	-	>250	>200	>150	>50	>50	>20	Vomiting, rhinorrhea, lethargy, sore throat
[16]	118	3rd (75)	6	84	81	8	-	7	8	Chest tightness, fatigue
[17]	13	1st (5) 2nd (3) 3rd (5)	-	8	5	1	1	-	1	-
[18]	51	3rd	26	27	31	-	14	-	-	Fatigue
[19]	16	3rd	-	12	0	0	-	-	-	-
[20]	1	22 w	-	1	1	-	1	-	1	Vaginal bleeding, abdominal pain
[21]	78	27–41 w	20	24	29	8	11	7	5	Anosmia, rhinorrhea
[22]	594 (including 6 w postpartum Women)	1st (77) 2nd (241) 3rd (196) Postpartum (76)	-	71	119	-	71	-	-	Sore throat
[23]	1	38 w	-	-	1	-	-	-	-	Chest tightness
[6]	2	24 w, 27 w	-	2	0	1	-	0	-	-
[7]	15	37 w	-	10	6	-	-	-	1	-
[24]	1	34 w	-	-	-	1	1	-	-	-
[25]	1	38 w	-	1	1	-	-	1	1	Rhinorrhea, sore throat
[26]	1	22 w	-	1	-	-	-	-	-	Rhinitis
[27]	1	34 w	-	1	-	-	-	-	1	-
[28]	2	34 w, 37 w	-	1	1	1	-	-	1	-
[8]	30	30–40.9 w	8	11	5	-	-	-	-	Abdominal pain, haemoptysis, fatigue, poor appetite
[29]	1	33 w	-	-	-	-	-	-	-	-
[30]	1	32 w	-	1	-	-	-	-	-	Flu-like symptoms
[31]	1	38 w	-	1	-	-	-	-	-	-
[32]	64	29.9 ± 5.8 w	-	-	-	-	-	-	-	-
[33]	1	19 w	-	1	1	-	1	-	1	Sore throat, fatigue
[34]	38	29.3 ± 8.5	-	10	25	13	-	-	7	Sore throat, fatigue, anosmia
[35]	100	31.3 w (median)	-	62	80	30	26	-	10	Anosmia, sore throat
[36]	9	36–39 w	-	7	4	1	3	-	1	Sore throat, malaise
[37]	7	37–41 w	-	6	1	1	-	-	1	-
[38]	1	35 w	-	-	1	-	-	-	-	-
[39]	19	35–41 w	-	11	5	5	-	-	2	-
[40]	17	35–41 w	-	3	6	2	-	-	3	Nasal congestion, sputum production
[36]	1	35 w	-	1	-	1	-	-	-	Fatigue
[41]	3	34–38 w	-	2	3	1	-	-	-	-
[42]	1	35 w	-	-	1	-	-	-	-	-
[43]	1	39 w	-	-	1	-	-	-	-	-
[44]	1	29 w	-	1	-	1	-	-	-	Rhinitis
[45]	1	34 w	-	1	-	1	-	-	-	Nasal congestion
[46]	7	>36 w	-	7/7	6/7	-	-	-	6/7	-
[47]	4	3rd	-	3/4	2/4	2/4	2/4	-	-	Fatigue
[48]	1	30 w	-	1/1	-	-	-	-	-	-
[49]	9		-	9/9	9/9	-	-	-	1	-
[50]	7	28–37 w	2/7	2/7	3/7	-	3/7	2/7	-	Chest pain
[51]	1	35 w	-	1/1	1/1	-	-	-	-	-
[52]	1	38 w	1	-	-	-	-	-	-	-
[53]	1	33 w	-	1/1	-	1/1	-	-	-	-
[54]	9	24–36 w	-	9/9	9/9	5/9	4/9	-	-	-

**Table 2:** An overview of the reported neonatal outcomes in pregnant patients diagnosed with COVID-19 as described in the included studies.

Study	No of Pregnant Women	Completed Pregnancy	Vaginal Birth	C-Section	Preterm Delivery	Neonatal Adverse Outcomes	NICU Admission	Neonatal Death	Stillbirth	Miscarriage
[10]	1	1	1	0	1	0	1	0	0	0
[11]	1	1	0	1	1	SGA	1	1	0	0
[12]	1	1	0	1	1	Respiratory distress	1	0	0	0
[14]	1219	1196	-	450	204	-	254	5	-	-
[15]	427	266	106	156	66	Neonatal encephalopathy	67	2	3	4
[16]	118	68	5	63	14	-	-	0	0	-
[17]	1	1	-	1	-	-	-	-	-	-
[18]	13	6	1	4	2	Neonatal pneumonia	0	0	0	1
[19]	51	51	26	25	10	-	-	0	0	0
[20]	16	16	2	14	3	-	-	0	0	0
[21]	1	0	0	0	0	-	-	-	-	1
[75]	31	31	25	6	1	2 infected neonates	2	0	0	0
[22]	116	106	63	43	14	Prolonged QT syndrome, mild respiratory distress, short bowel syndrome, tachycardia	12	0	0	0
[6]	1	1	1	-	-	-	1: quarantine	-	-	-
[71]	65	65	13	52	9	Asphyxia, fever, diarrhea	-	-	-	-
[7]	2	2	1	1	0	Jaundice	0	0	0	0
[24]	15	15	1	14	1	NRDS	15: quarantine	0	0	0
[25]	1	1	-	1	1	-	1	0	0	0
[26]	1	1	-	1	-	Severe COVID-19	1	0	0	0
[27]	1	1	1	-	2	-	-	-	-	2 (twins)
[28]	1	1	1	-	1	Jaundice, fever, respiratory distress hypoxia	1	0	0	0
[8]	2	2	-	2	1	-	-	-	-	-
[29]	30	30	7	23	5	-	-	-	-	-
[30]	1	1	1	-	1	Intubation	1	-	-	-
[31]	1	1(triplets)	-	3	3	1: NCPAP	3	0	0	0
[32]	1	1	1	-	-	Abdominal distension, respiratory acidosis, intubation	1	0	0	0
[33]	64	32	8	24	29	2: IUGR	21	0	0	0
[34]	1	1	1	-	-	-	-	-	-	1
[35]	38	17	10	7	10	3: intubated	3	0	0	1
[36]	100	33	17	16	20	6: intubated	10	1	0	0
[36]	9	9	0	9	4	-	0	0	0	0
[37]	7	7	0	7	0	1: mild pulmonary infection	0	0	0	0
[38]	1	1	-	1	1	-	0	0	0	0
[39]	19	19	1	18	0	-	19: isolation	0	0	0
[40]	17	17	0	17	3	5: neonatal pneumonia		0	0	0
[36]	1	1	-	1	1	Tachypnea, moaning, periodic breath	1	0	0	0
[41]	3	3	3	0	1	-	0	0	0	0
[42]	1	1	0	1	1	-	0	0	0	0
[43]	1	1	0	1	0	-	0	0	0	0
[44]	1	1	0	1	1	-	1	0	0	0
[45]	1	1	0	1	0	-	1: quarantine	0	0	0
[46]	7	7	0	7	4	Respiratory distress	5	0	0	0
[47]	4	4	1	3	0	TTN, rash	2	0	0	0
[48]	1	1	0	1	1	-	1: isolation	0	0	0
[49]	9	9	2	7	6	Dyspnea, fever, vomit, NRDS, pneumothorax, thrombopenia		1	0	0
[51]	1	1	0	1	1	Intubation, neurological symptoms	1	0	0	0
[52]	1	1	0	1	0	-	0	0	0	0
[53]	1	1	0	1	1	Intubation	1	0	0	0
[54]	9	9	1	8		Intubation, pneumonia		2	4	-

**Table 3:** An overview of the reported evidence on vertical transmission in pregnant patients diagnosed with COVID-19 as described in the included studies.

Study	Neonatal Throat Swab (+)	Amniotic Fluid (+)	Vaginal Secretions (+)	Placenta (+)	Breastmilk Viral RNA (+)	IgM (+)	IgG (+)	Cord Blood Viral RNA (+)	IgM (+)	IgG (+)	Neonatal Serum IgM (+)	IgG (+)	Other (+)
[83]	0/1	-	-	-	-	-	-	-	-	-	-	1/1	
[11]	-	-	-	1/1	-	-	-	-	-	-	-	-	Umbilical cord
[12]	1/1	1/1	-	-	-	-	-	-	-	-	-	-	
[81]	-	0/1	-	0/1	-	-	-	-	-	-	-	-	
[78]	1/1	-	-	-	-	-	-	-	-	-	1/1	1/1	
[79]	-	1/1	-	1/1	-	-	-	-	-	-	-	-	Fetal membranes
[82]	-	-	-	2/2	-	-	-	-	-	-	-	-	Fetal lungs and kidneys
[15]	12/240	-	-	-	-	-	-	-	-	-	-	-	
[16]	0/8	-	-	-	0/3	-	-	-	-	-	-	-	
[17]	-	0/1	-	0/1	0/1	1/1	1/1	0/1	1/1	1/1	-	-	
[18]	0/5	0/9	0/13	0/9	1/3	-	-	-	-	-	1/5	1/5	
[20]	0/3	-	-	-	-	-	-	-	-	-	-	-	
[21]	-	-	-	1/1	-	-	-	-	-	-	-	-	Umbilical cord
[75]	2/31	0/3	1/30	2/31	1/11	1/10	0/10	1/30	1/30	12/30	-	-	
[22]	0/120	-	-	-	-	-	-	-	-	-	-	-	
[6]	0/1	-	0/1	-	0/1	0/1	1/1	-	-	-	0/1	1/1	
[71]	0/38	-	-	-	-	-	-	-	-	-	-	-	
[7]	0/2	-	-	-	-	-	-	-	-	-	0/2	2/2	
[24]	0/15	0/15	-	0/15	-	-	-	-	-	-	-	-	
[25]	1/1	-	-	-	-	-	-	-	-	-	-	-	
[26]	1/1	-	-	-	1/1	-	-	-	-	-	-	-	Infant’s and mother’s stool sample
[27]	-	2/2	-	2/2	-	-	-	-	-	-	-	-	Maternal Blood sample
[28]	1/1	-	-	-	-	-	-	-	-	-	-	-	
[8]	0/2	0/2	-	1/2	1/2	-	-	1/2	-	-	-	-	
[29]	0/30	-	-	-	-	-	-	-	-	-	-	-	
[30]	0/1	0/1	-	0/1	0/1	-	-	-	-	-	-	-	
[31]	3/3	-	-	-	-	-	-	-	-	-	-	-	
[32]	1/1	-	-	-	-	-	-	-	-	-	-	-	Tracheal aspiration, anal swab
[33]	1/33	-	-	-	-	-	-	-	-	-	-	-	
[34]	-	0/1	0/1	1/1	-	-	-	-	-	-	-	-	
[36]	1/36	-	-	-	-	-	-	-	-	-	-	-	
[36]	0/6	0/6	-	-	0/6	-	-	0/6	-	-	-	-	
[37]	1/3	-	-	0/1	-	-	-	0/1	-	-	-	-	
[38]	0/1	0/1	-	0/1	0/1	-	-	0/1	-	-	-	-	
[39]	0/19	0/19	-	-	0/10	-	-	0/19	-	-	-	-	
[40]	2/17	-	-	-	-	-	-	-	-	-	-	-	
[36]	0/1	0/1	0/1	0/1	0/1	-	-	0/1	-	-	-	-	
[41]	0/3	-	-	-	-	-	-	-	-	-	-	-	
[42]	0/1	0/1	-	0/1	0/1	-	-	0/1	-	-	-	-	
[43]	0/1	-	-	-	-	-	-	-	-	-	-	-	
[45]	0/1	-	0/1	-	0/1	-	-	-	-	-	1/1	1/1	
[46]	0/6	0/5	-	-	-	-	-	0/5	-	-	-	-	
[47]	0/3	-	-	-	-	-	-	-	-	-	-	-	
[48]	0/1	0/1	-	0/1	-	-	-	0/1	-	-	-	-	
[49]	0/9	-	-	-	-	-	-	-	-	-	-	-	
[51]	1/1	1/1	1/1	1/1	-	-	-	-	-	-	-	-	Rectal swab, neonatal blood
[52]	0/1	-	-	1/1	-	-	-	-	-	-	0/1	0/1	
[53]	1/1	-	-	-	-	-	-	-	-	-	0/1	0/1	

## Data Availability

Not applicable.
